# A Novel NanoMIP–SPR Sensor for the Point-of-Care Diagnosis of Breast Cancer

**DOI:** 10.3390/mi14051086

**Published:** 2023-05-21

**Authors:** Kadir Erol, Gauri Hasabnis, Zeynep Altintas

**Affiliations:** 1 Institute of Materials Science, Faculty of Engineering, Kiel University, 24143 Kiel, Germany; ker@tf.uni-kiel.de (K.E.); gha@tf.uni-kiel.de (G.H.); 2 Environmental Health Program, Department of Medical Services and Techniques, Vocational School of Health Services, Hitit University, Corum 19030, Turkey; 3 Kiel Nano, Surface and Interface Science (KiNSIS), Kiel University, 24118 Kiel, Germany

**Keywords:** SPR sensor, human epidermal growth factor receptor 2 (HER2), peptide imprinting, nanoMIPs, breast cancer diagnosis

## Abstract

Simple, fast, selective, and reliable detection of human epidermal growth factor receptor 2 (HER2) is of utmost importance in the early diagnosis of breast cancer to prevent its high prevalence and mortality. Molecularly imprinted polymers (MIPs), also known as artificial antibodies, have recently been used as a specific tool in cancer diagnosis and therapy. In this study, a miniaturized surface plasmon resonance (SPR)-based sensor was developed using epitope-mediated HER2-nanoMIPs. The nanoMIP receptors were characterized using dynamic light scattering (DLS), zeta potential, Fourier-transform infrared spectroscopy (FT-IR), transmission electron microscopy (TEM), energy-dispersive X-ray spectroscopy (EDX), and fluorescent microscopy. The average size of the nanoMIPs was determined to be 67.5 ± 12.5 nm. The proposed novel SPR sensor provided superior selectivity to HER2 with a detection limit (LOD) of 11.6 pg mL^−1^ in human serum. The high specificity of the sensor was confirmed by cross-reactivity studies using P53, human serum albumin (HSA), transferrin, and glucose. The sensor preparation steps were successfully characterized by employing cyclic and square wave voltammetry. The nanoMIP–SPR sensor demonstrates great potential for use in the early diagnosis of breast cancer as a robust tool with high sensitivity, selectivity, and specificity.

## 1. Introduction

Breast cancer (BC) is considered one of the leading causes of death in females, and the BC-caused mortality rate has been increasing annually [1,2]. Despite the high prevalence and mortality caused by BC, its early recognition and diagnosis can significantly increase the survival rate. Hence, it is necessary to develop susceptible innovative methods for preventive and therapeutic measures to increase the survival rate of patients with BC [2]. Human epidermal growth factor receptor-2 (HER2) is a transmembrane tyrosine kinase receptor and plays a vital role in regulating average cell growth, differentiation, and survival [3,4]. The overexpression of HER2 is associated with a molecular anomaly in 15–25% of patients with BC. Accordingly, HER2 is considered a prognostic and predictive biomarker for the detection and monitoring of breast cancer [5]. 

The expression of HER2 has been recently assessed on the basis of a set of invasive techniques, such as immunohistochemistry (IHC) and biopsy using fluorescent in situ hybridization (FISH) [6,7,8,9]. The most important disadvantages of these methods are difficulties due to their complex nature and multi-step procedures [10], high cost, the necessity of the long-term assessment of high-quality tissue samples [11,12], and the requirement of trained personnel. Enzyme-linked immunosorbent assay (ELISA) is generally used to detect HER2 in the serum. However, ELISA has some drawbacks, such as suppressing thermodynamic/kinetic studies of antibody–antigen interaction and using labeled molecules to induce false-positive responses [13]. Therefore, there is still a strong need to develop a rapid, easy-to-use, noninvasive, inexpensive, and ultra-sensitive method for detecting the HER2 biomarker with the purpose of minimizing the technical impediments of conventional methods. Such a method may also accurately identify HER2 in the bloodstream [1]. 

To date, various electrochemical, SPR, piezoelectric, and FRET-based optical biosensors that are cheaper and more sensitive than serological methods have been reported for the detection of HER2 [1]. These biosensors enable the selective and fast detection of HER2 with a low detection limit [14]. However, the materials used as bio-receptors, including aptamers [14], antibodies [15], and peptides [16], possess some disadvantages, such as being unstable and expensive and having a short shelf-life, leading to difficulties in integrating biosensor systems. 

Recently, molecularly imprinted polymers (MIPs), possible alternatives to natural recognition elements, have increasingly been used in biosensing and have outstanding advantages, such as bearing highly specific, sensitive, stabile biorecognition cavities on the sensor surfaces; robustness; resistance to extreme physical conditions; and being synthesized via relatively simple, cheap, and scaleable protocols [17,18]. In the molecular imprinting field, epitope imprinting is generally preferred over whole-protein imprinting because of the problems arising from the large, complex structure of proteins as well as the changes in their conformation during the imprinting process [19,20]. Epitope-imprinted MIPs are able to recognize both the epitope and whole macromolecules (e.g., proteins, viruses, and bacteria) and bind to them specifically [21]. The solid-phase synthesis approach for obtaining nanoMIPs results in the formation of high-affinity and -sensitivity receptors even in aqueous media, which is the natural environment of biological molecules. In addition to this, it allows low detection limits for developed sensors [22]. NanoMIPs are promising alternatives to natural antibodies in diagnostic and in vivo applications due to their cost efficiency, high affinity, and stability [23,24,25]. 

To our knowledge, in this study, a miniaturized SPR-based sensor was developed for the first time for the detection of HER2 using epitope-mediated nanoMIPs. The nanoMIP receptors were characterized using dynamic light scattering (DLS), zeta potential, Fourier-transform infrared spectroscopy (FT-IR), transmission electron microscopy (TEM), energy-dispersive X-ray spectroscopy (EDX), fluorescence microscopy analyses, and electrochemical methods. The nanoMIP–SPR sensor could detect HER2 with high selectivity, specificity, and sensitivity.

## 2. Materials and Methods

### 2.1. Reagents and Chemicals

N-isopropylacrylamide (NIPAm), N,N′-methylenebisacrylamide (BIS), N-(3-aminopropyl) methacrylamide hydrochloride (APMA), acrylic acid (AAc), N-tert-butylacrylamine (TBAm), N,N,N′,N′-tetramethylethylenediamine (TEMED), ammonium persulphate (APS), ethanol (absolute), methanol (ACS reagent, ≥99.8%), acetone (ACS reagent, ≥99.5%), toluene (anhydrous), glutaraldehyde (GA), ethanolamine, Tween 20 (polyoxyethylenesorbitan monolaurate), phosphate-buffered saline (PBS), sulfuric acid, hydrogen peroxide, 11-mercaptoundecanoic acid (MUDA), N-hydroxysuccinimide (NHS), 1-ethyl-3-(3-dimethylaminopropyl) carbodiimide (EDC), sodium hydroxide (NaOH), sodium borohydride (SBH), transferrin from human blood plasma, albumin from human serum (HSA), and tumor protein p53 were purchased from Sigma Aldrich Chemical Co. (Hamburg, Germany). 3-Aminopropyltriethoxysilane (APTES) was obtained from Fisher Scientific (Schwerte, Germany). Methacryloxyethyl thiocarbamoyl rhodamine B was provided by Polysciences Europe (Bergstrasse, Germany). HER2-ECD (10004-HCCH) and the peptide (163–175: DTNRSRACHPCSP) chosen for imprinting were provided by Sino Biological (Eschborn, Germany) and GenScript Biotech (Nanjing, China), respectively. Glass beads (0.75–1.0 mm), glucose, and syringe filters (Rotilabo PTFE, 0.45 µm and 0.22 µm) were supplied by Carl Roth (Karlsruhe, Germany). Without additional purification, all compounds and solvents were of analytical or HPLC quality. A 0.22 µm syringe filter was used to filter a phosphate-buffered saline + 0.05% Tween (PBS/T) buffer. Double-distilled water (DDW, produced by Ion Ex Mischbettpatrone, Arno Willers, Hamburg, Germany) was used to prepare all solutions in the aquatic environment.

### 2.2. Preparation of Glass Beads for NanoMIP Synthesis

**Figure 1:**
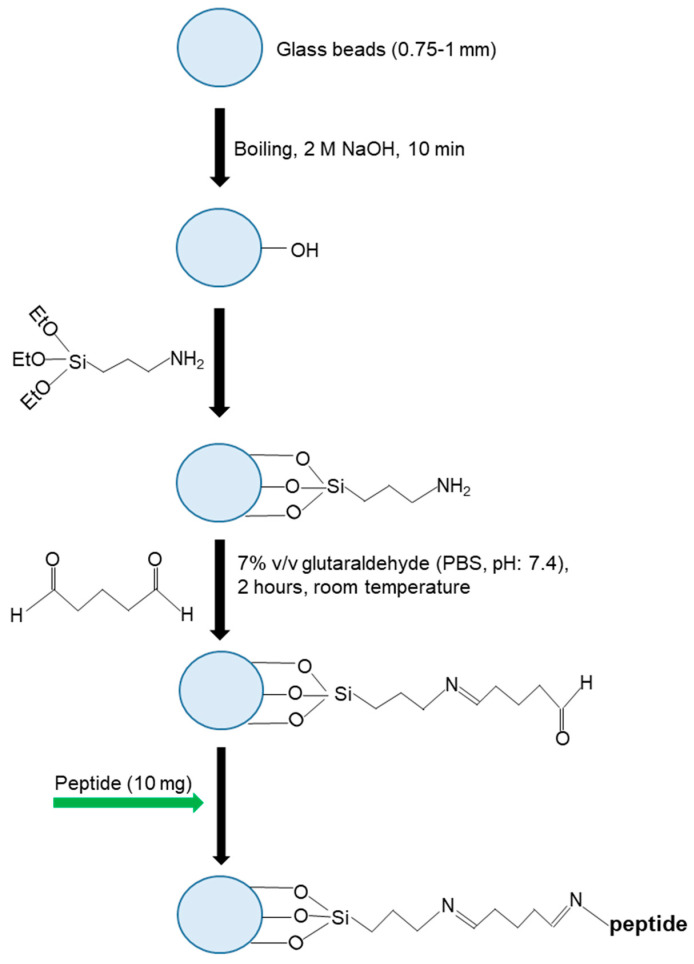
The immobilization of the HER2-peptide on glass beads as the template.

First, the glass beads, the firm support of the solid-phase synthesis, were prepared for the silanization process. For this, 60 g of glass beads was weighed in a beaker and activated by boiling in 2 M NaOH solution for 15 min. Then, the glass beads were washed five times with DDW, four times with PBS (pH: 7.4), and five times with DDW. After the last wash with excess water, the pH value of the wash water was determined to be 7.0–7.5, and the process was continued by washing the glass beads twice with acetone. The beads were then dried in a nitrogen atmosphere and incubated overnight in 2% v/v APTES solution (in anhydrous toluene) in a sealed container. After this step, the silanization part of the process was completed. The glass beads were removed from the APTES solution and washed four times with acetone and methanol. Beads dried with nitrogen gas were incubated in a solution of 7% GA prepared in PBS (pH: 7.4) for 120 min. This is an intermediate step so the peptide can be covalently attached to the glass beads. Following this step, 10 mg of the cold peptide solution dissolved in 40 mL PBS was added to the glass beads, which were washed five times with DDW. This is an essential step in the process, and overnight incubation was allowed to ensure that the peptide was covalently bound to the beads. After incubation, the peptide solution was poured out, and the glass beads were washed five times with DDW. Afterward, the glass beads were treated with SBH solution (1 mg mL^−1^) prepared in PBS (pH: 7.4) for 30 min. SBH is an effective aldehyde-blocking agent with a strong reducing structure [22]. Glass beads, repeatedly washed with an excess of DDW, interacted with 50 mL of 0.1 mM ethanolamine (pH: 7.4 in PBS) solution for 15 min to avoid non-specific interactions and self-reaction of unconjugated free GA groups. After the formed interaction, the ethanolamine solution was poured out, and the glass beads were washed five times with DDW, dried in a nitrogen atmosphere, and placed in the reaction flask. After this step, the glass beads were ready for nanoMIP synthesis (Figure 1). 

### 2.3. Synthesis of Target NanoMIPs

**Figure 2:**
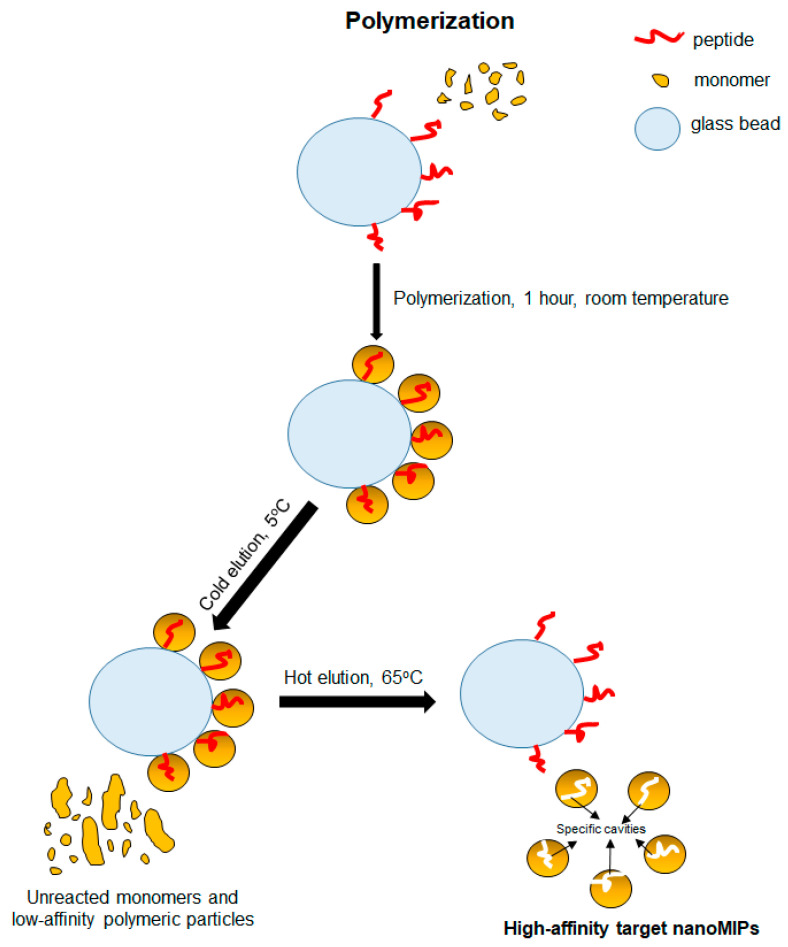
The synthesis principle of peptide-imprinted nanoMIPs.

The necessary monomers for polymerization were first mixed. In brief, 39 mg of NIPAm, 2 mg of BIS, 54 mg of APMA, and 2.2 µL of AAc were added to 98 mL of PBS (pH: 7.4) solution in a reaction flask. In addition, 38 mg of TBAm and 3 mg of methacryloxyethyl thiocarbamoyl rhodamine B monomers were separately dissolved in 1 mL of absolute ethanol and added to the monomer mixture in the reaction flask. The monomer mixture was stirred under a magnetic stirrer for 30 min. Afterward, the mixture was sonicated (Transsonic Digital S, Elma Schmidbauer GmbH, Singen, Germany) for 20 min, and then nitrogen gas was passed through the mixture for 20 min. The process was continued by adding peptide-coupled glass beads to the reaction flask, and nitrogen gas was re-passed via the mixture for 2 min. Then, 800 µL of APS solution (initiator, 60 mg mL^−1^, in water) and 24 µL of TEMED (activator) were instantly added to the monomer mixture, nitrogen gas was passed through the mouth of the reaction flask for 30 s, and the flask was tightly closed with a screw cap. Polymerization was completed in 1 h, the bottle was opened, and the cold wash and hot wash stages were carried out one after the other. Hot and cold washing steps were applied in a polypropylene SPE tube (including polyethylene frits: 20 μm pore size). The cold wash process was performed to remove unreacted monomers and low-affinity nanoMIPs from the polymerization medium. For this step, three consecutive washes were carried out with 20 mL of DDW (5 °C). Then, the SPE column containing the glass beads was kept in a water bath (GFL Shaking Water Bath 1083, Burgwedel, Germany) for 15 min to prepare for hot washing. In the next step, seven washes were performed with 20 mL of DDW (65 °C) each to collect the high-affinity (target) nanoMIPs (Figure 2). The collected nanoMIP pool (140 mL) was stored at 4 °C for the subsequent experimental studies. Three samples of 10 mL were taken to calculate the yield from the obtained nanoMIP suspension. These samples were placed in the predetermined weight glass vials and dried using freeze-drying (ALPHA 2–4LD Plus freeze-dryer, Christ, Osterode am Harz, Germany) for 48 h. After completely removing the water through the drying process, the glass vials were re-weighed, and the weight of the nanoMIPs was determined by subtracting the tare of the vials from the obtained weight values.

### 2.4. Characterization of NanoMIPs

The size distribution profiles of nanoMIPs were determined by a dynamic light scattering (DLS) device (Malvern Panalytical, Zetasizer Pro., Herrenberg, Germany). Sixty runs were performed in the backscattered mode for each recording. In addition, the zeta potential value was determined with the same device to measure the stability of the nanoMIPs in water. The sample of the nanoMIP solution, which was dropped on the glass slider and dried, was imaged with a fluorescence microscope (BZ-X800LE, Keyence, Neu-Isenburg, Germany). The microscope (BZ-PA10, Plan Apochromat 10X, NA 0.45, WD 4 mm) had a 40 W LED fluorescent light source and a power supply of 100 to 240 VAC ± 10%, 50/60 Hz. Of note, the fluorescent property of methacryloxyethyl thiocarbamoyl rhodamine B monomer (excitation max: 548 nm, concentration in the polymerization mixture: 45 µM) gave the polymer a fluorescent nature. An FT-IR device (Cary 630 FTIR, Agilent Technologies, Santa Clara, CA, USA) was used to analyze the functional groups that existed in nanoMIPs. TEM analyses were performed on an FEI Tecnai F30 G2 STwin (300 kV, FEG) equipped with an EDX detector (Si/Li, EDAX) to visualize the morphological structure of the nanoMIPs and analyze the elemental composition of the polymer. 

In addition, the successful development of the nanoMIP sensor was verified using two main electrochemical techniques, including cyclic voltammetry (CV) and square-wave voltammetry (SWV). For this, a gold substrate was used as the working electrode in the electrochemical measurement setup (PalmSens4 workstation, Belltec, Lüdenscheid, Germany). All CV measurements were performed at a potential range of −0.2 to 0.8 V and a scan rate of 0.05 V s^−1^. The range of applied potentials for SWV measurements was −0.3 to 0.8 V at an amplitude of 0.05 V and a frequency of 5 or 10 Hz. The experiments were performed at room temperature.

### 2.5. Optical Detection of the HER2 Biomarker

The bare gold SPR chip was initially cleaned with a mixture of hydrogen peroxide (35%, 2 mL), ammonia (25%, 2 mL), and millipore water (50 mL) and boiled at 80 °C for 20 min. For this process, two gold chips were placed in a chip holder and immersed in the preheated mixture. Most contaminants on the gold surface were eliminated using piranha solution (a 3:1 mixture of sulfuric acid and 30% hydrogen peroxide). After this treatment, the gold chips were washed five times with millipore water and three times with absolute ethanol. The chips were then dried with a gentle flow of nitrogen gas. Next, the gold chips were immersed in the MUDA solution (2 mM, 5 mL) prepared in absolute ethanol in a Petri dish and incubated overnight in the dark. This process created a self-assembled monolayer on the surface of the chips. After incubation, the gold chips were washed with an excess of absolute ethanol and double-distilled water, gently dried with nitrogen gas, and stored in a fridge at 4 °C until the time of use. 

A miniaturized angular SPR device (CORGI IIF, Plasmetrix, Montreal, QC, Canada) was used for all detection studies, including HER-2 peptide and protein as the target molecules. During the sensor experiments, the solutions were allowed to pass through the chip surface with a peristaltic pump (Ismatec Reglo ICC Digital pump, 2-channel, Cole-Parmer GmbH, Wertheim, Germany), providing a flow rate of 4 µL min^−1^. MUDA-coated chips were activated with a 4 min injection of a freshly made EDC/NHS solution (0.4 M EDC, 0.1 M NHS) for the covalent immobilization of the nanoMIPs with the aid of amine coupling chemistry. A constant flow rate (4 µL min^−1^) was maintained for 8 min to immobilize the nanoMIPs on the gold chip surface. To prepare the nanoMIP medium (500 µg mL^−1^), the suspension was prepared with degassed PBS/T and filtered through a 0.22 µm syringe filter prior to sonication for 30 min. 

**Figure 3:**
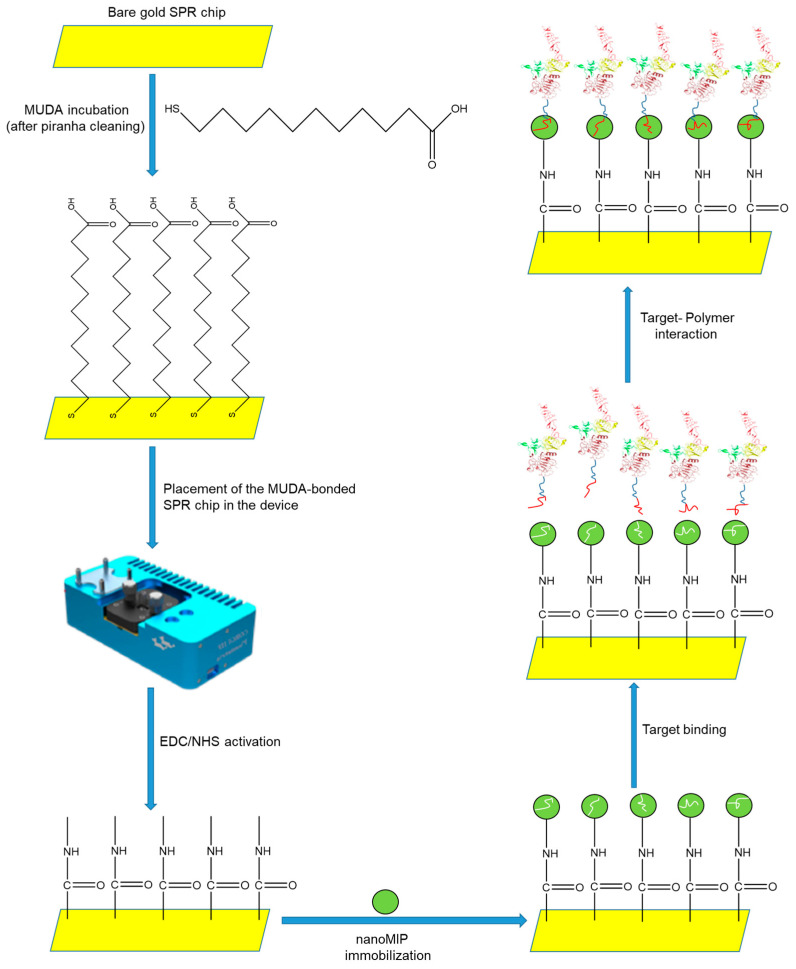
The preparation of nanoMIP-based sensor system.

The samples were prepared in PBS/T to promote fluid flow through the sensor and microtube channels and prevent air from becoming trapped inside the microfluidics. The nanoMIP suspension was then re-filtered with a 0.45 µm syringe filter. The chip surface then interacted with 1.0 mM ethanolamine solution for 4 min to block possible active sites that may have remained on the surface after nanoMIP immobilization. The injection of samples, including HER2 peptide and HER2 biomarker, at varying concentrations were subsequently carried out (Figure 3). The association and dissociation times were set at 5 and 2 min, respectively. 

For selectivity studies, a control nanoMIP was obtained by imprinting a different peptide (ISASRKLQLK). It was immobilized on the sensor surface by following the aforementioned procedure prior to the injection of target analytes for the determination of sensor selectivity. Furthermore, the specificity of the developed nanoMIP–SPR sensor was realized by studying reference molecules (i.e. P53, HSA, transferrin, and glucose).

## 3. Results and Discussion

### 3.1. Size and Stability of Target NanoMIPs

**Figure 4:**
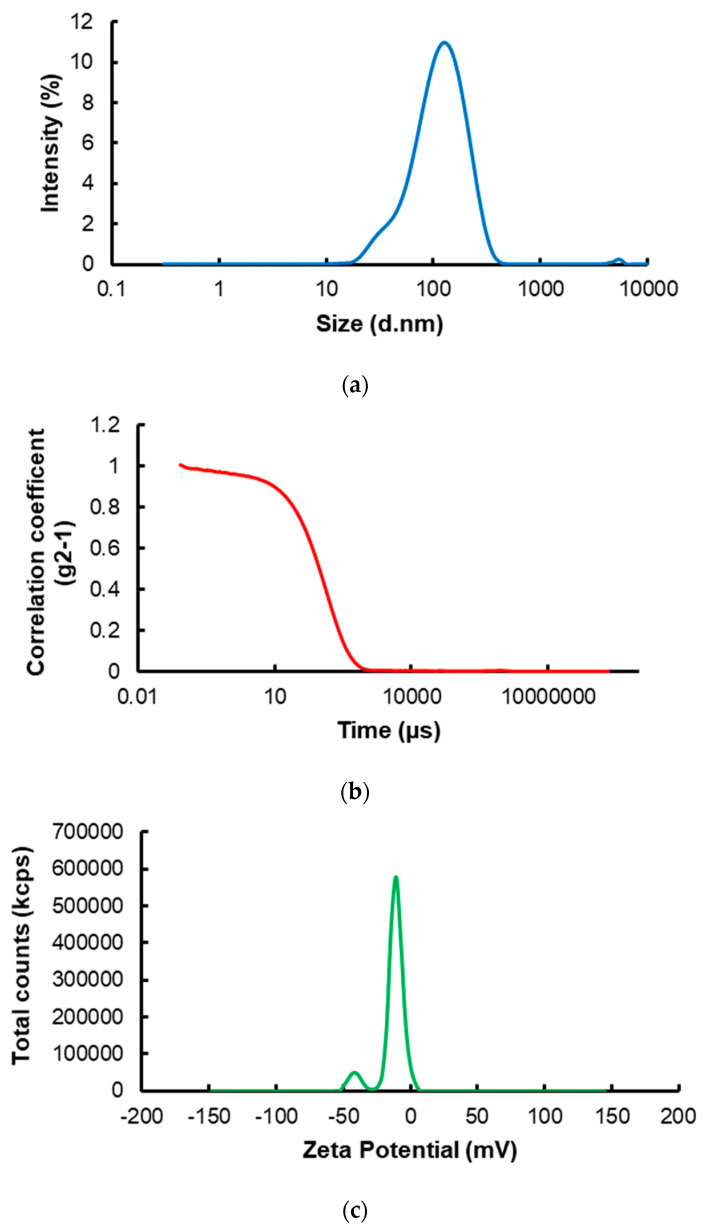
(**a**) The hydrodynamic size distribution of target nanoMIPs; (**b**) The correlation fit of DLS analysis; (**c**) The zeta potential profile of nano-polymers.

The hydrodynamic size of target nanoMIPs was measured in DDW at room temperature by employing DLS. The nanoMIPs were discovered to have an average hydrodynamic radius of 97.79 ± 0.53 nm and a polydispersity index (PDI) of 0.263, revealing remarkably uniform and monodisperse particles (Figure 4a,b). Additionally, the zeta potential of the nanoMIPs in DDW was tested to ascertain the stability of the solution. The average zeta potential of nanoparticles (NPs) was found to be −12.37 ± 0.32 mV (Figure 4c). It was hypothesized that the NPs forming a dispersed phase endowed the solution with a colloidal character. 

### 3.2. Fluorescence Microscopy and TEM Analyses

**Figure 5:**
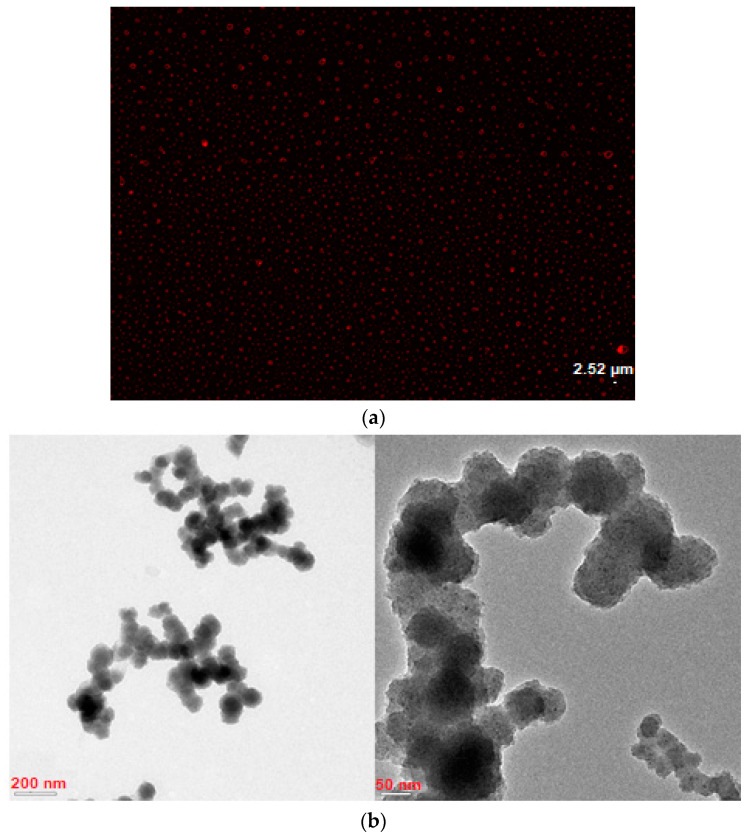
(**a**) Fluorescence microscopy and (**b**) TEM images of nanoMIPs.

For fluorescence microscopy imaging, a certain concentration (500 µg mL^−1^) of nanoMIP solutions was prepared. The fluorescence microscopy image proved that the fluorescent monomer (methacryloxyethyl thiocarbamoyl rhodamine B) used in the synthesis of nanoMIP was well incorporated into the polymeric structure (Figure 5a). Additionally, TEM images confirmed the exact size, shape, and uniformity of the nanoMIPs. (Figure 5b). The size of particles acquired by TEM was approximately 67.5 ± 12.5 nm which was smaller than those measured by DLS due to solvation and swelling of polymer particles in the latter case. Another reason is that the agglomeration of nanoMIPs in solution causes an evident size increment in DLS measurements [26]. 

### 3.3. FT-IR and EDX Analysis

**Figure 6:**
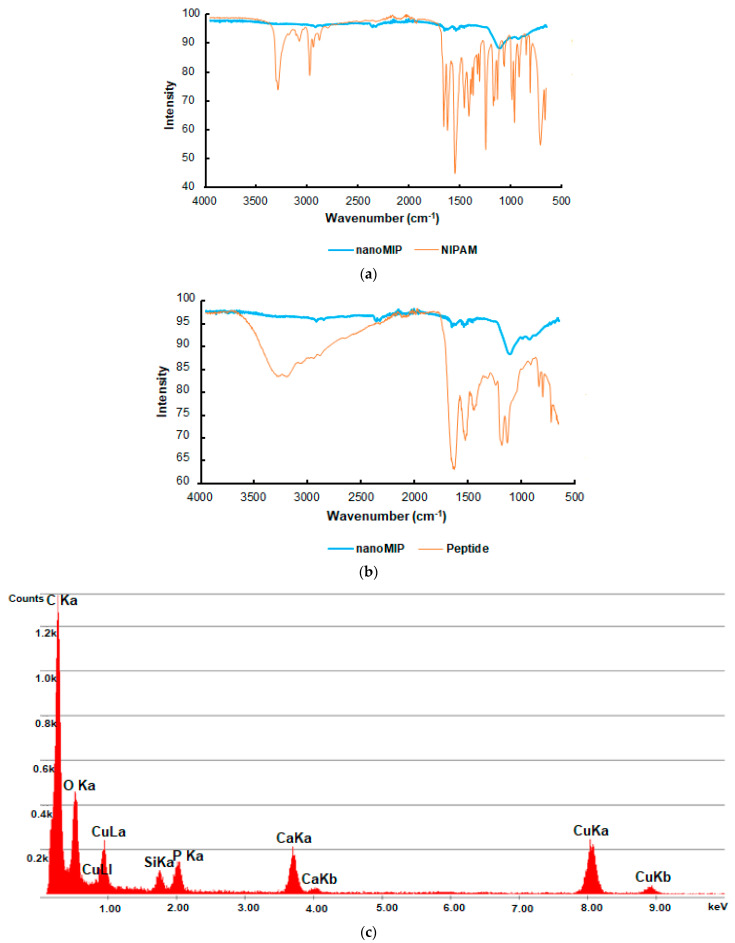
The comparison of FT-IR spectrum of nanoMIPs with FT-IR spectrum of (**a**) NIPAM; (**b**) imprinted peptide; (**c**) the elemental composition of nanoMIPs.

FT-IR analysis was performed to prove the successful synthesis of peptide-imprinted nanoMIPs (Figure 6a,b). The results revealed that the spectrum of NIPAM as a structural monomer was significantly different from the polymer (nanoMIP) spectrum and exhibited only a few expected peaks. C-H asymmetric stretching (2967 cm^−1^), C-H symmetric stretching (2877 cm^−1^), C=O amide group (1632 cm^−1^), N-H bending (1545 cm^−1^), C-N stretching (1366 cm^−1^), -CH_2_ bending (1452 cm^−1^), and -CH_3_ bending (1385 cm^−1^) vibrations were notable peaks in the spectrum of NIPAM. Vibrations such as C-H asymmetric–symmetric stretching (2917 cm^−1^ and 2848 cm^−1^, respectively), C=O amide group (1636 cm^−1^), and N-H bending (1539 cm^−1^) also appeared in the polymer spectrum. In addition, the FT-IR analysis of the imprinted peptide revealed that no peak of the peptide was identified in the nanoMIP spectrum. This indicates that the template removal from the polymeric structure, as one of the most critical steps of the imprinting process, was effectively achieved. 

Furthermore, the EDX analysis was performed to analyze the elemental composition of nanoMIPs. Carbon and oxygen elements in the structure indicated the synthesis of the desired organic polymer. (Figure 6c).

### 3.4. Electrochemical Characterization of NanoMIP-Based SPR Sensor

Square-wave voltammetry (SWV) and cyclic voltammetry (CV) are two widely used electrochemical analytical techniques to examine the surface changes at a working electrode (Au). The electrochemical analysis of the nanoMIP–SPR sensor was carried out using a three-electrode setup with counter (CE), reference (RE), and working (WE) electrodes made of Pt, Ag/AgCl, and Au, respectively [27]. By submerging the gold electrode in a redox marker solution (10 mM K_3_Fe(CN)_6_ in 0.1 M KCl), the sensor preparation steps were characterized using CV and SWV. 

**Figure 7:**
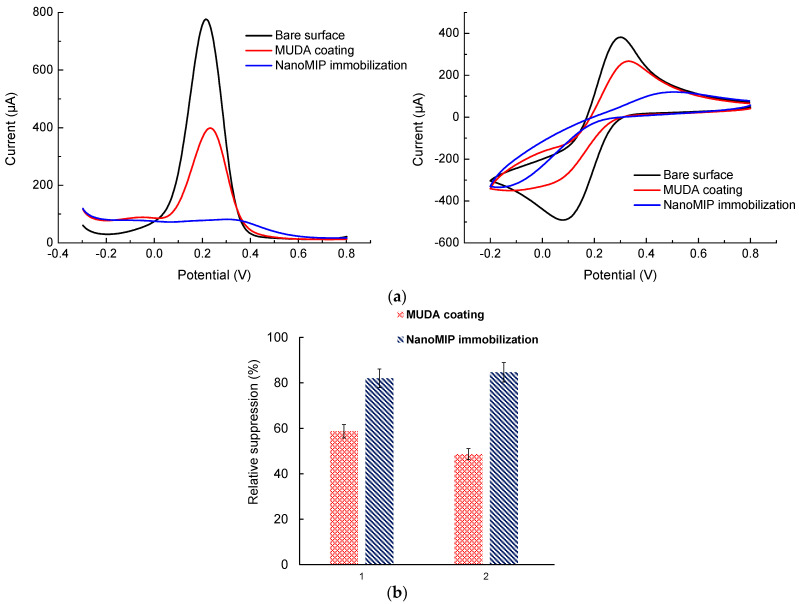
Electrochemical experiments with nanoMIPs. (**a**) SWV and CV graphs; (**b**) Relative suppression graphs (*n* = 3).

In Figure 7a, the bare Au surface, MUDA coating, and immobilized nanoMIPs are shown by CV and SWV curves. The highest current peak (black curve) corresponds to the bare surface, as it could freely participate in the electron exchange process with the redox marker solution. Due to the non-conductive MUDA layer on the Au surface, the current (red curve) was sufficiently decreased, which corresponds to the second-highest peak. The nanoMIPs particles covered the entire electrode surface, which is shown by the lowest current peak (blue curve). An excessive hindrance in electron exchange occurred between the redox marker and the nanoMIPs, and the current was massively decreased, which is indicated by both CV and SWV curves. 

Using SWV measurement, the relative suppression percentage with respect to the bare signal was calculated for MUDA and the nanoMIPs (Figure 7b). This percentage significantly increased for both sensor fabrication steps. Hence, the electrochemical characterization results confirmed the successful MUDA coating as well as nanoMIP immobilization on the gold surface. Of note, the characterization studies on two individual gold substrates provided very similar electronic signals, demonstrating the reproducible coating of MUDA and nanoMIPs on the sensing surfaces (Figure 7b). 

### 3.5. HER2 Detection and Kinetic Data Analysis

**Figure 8:**
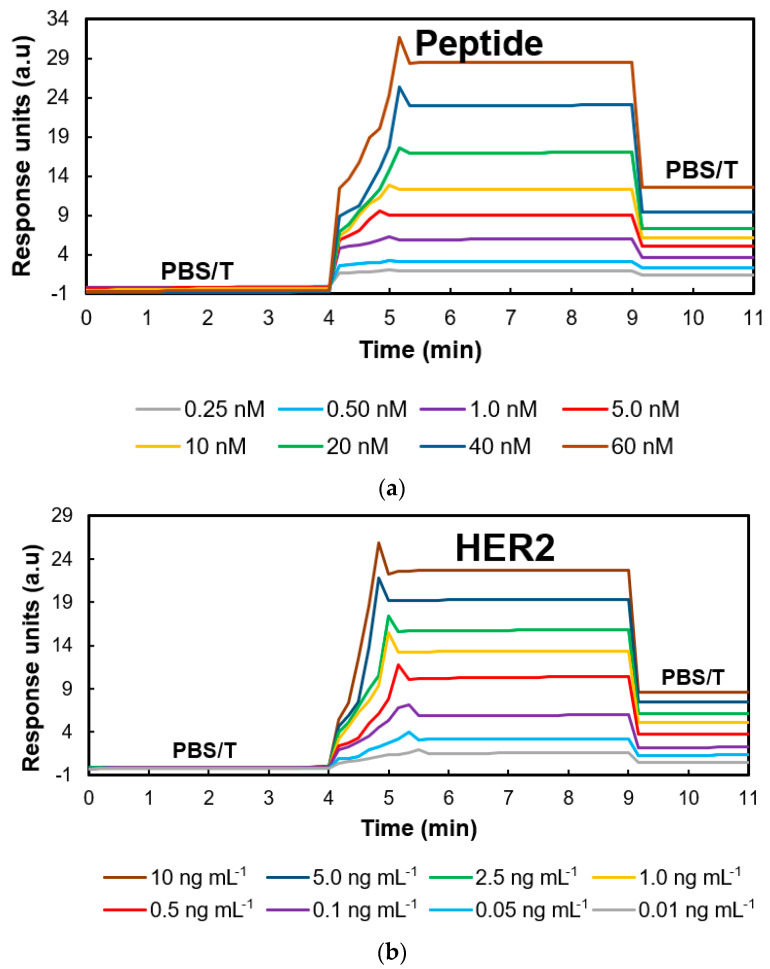
Sensograms for the real-time detection of (**a**) peptide and (**b**) HER2 in the PBST buffer.

Detection studies with the nanoMIP-based SPR sensors hierarchically began with a peptide analysis. As a result of the procedures described in Section 2.5, it was observed that the corresponding sensor signals increased gradually with increasing peptide concentration (Figure 8a). For these experiments, the peptide solutions prepared in PBS (pH: 7.4) were used at a concentration range of 0.25–60 nM. In addition, a graph was plotted against the SPR signal based on the peptide concentration to calculate the LOD value, which was expressed as the analyte concentration corresponding to the sample blank value plus three standard deviations. This value was determined to be 0.086 nM. 

The samples for HER2 detection were prepared in a similar way in PBS in the concentration range of 0.01–10 ng mL^−1^, and their stepwise injections onto the sensor’s surface from the lowest to highest concentration resulted in a gradual increase in the electronic signal (Figure 8b). The nanoMIP–SPR sensor revealed an LOD value of 3.8 pg mL^−1^. In experiments in which a 5 min injection time was sufficient for the association of the peptide as well as the protein, a 2 min interaction with the PBS/T solution provided the dissociation of these species bound to the surface. Thus, the high affinity of the epitope-mediated nanoMIPs was realized in a real-time manner. 

Furthermore, kinetic data analysis was performed to determine the dissociation constant (*K_D_*) by subjecting the sensor results to three major binding isotherms (i.e., Langmuir, Freundlich, and Langmuir–Freundlich). In theory, homogeneity (one-to-one) and heterogeneity (one-to-many) are realized with the Langmuir and Freundlich binding models, respectively. Although the Langmuir–Freundlich model combines two isotherms of adsorption and assumes a homogenous surface, the biosorption phenomenon is a cooperative process outlined by the following equation (Equation (1)).

(1) $$N=N_{m}KC^{n}/(1+KC^{n})$$

Herein, *K* and *n* stand for the isotherm constant and homogeneity index, respectively. The concentrations were significantly based on this model. While the model approaches the Freundlich model toward low concentrations, a trend consistent with the Langmuir model is observed at higher concentration values. By modifying the *N_t_*, *a*, and *m* parameters, where *a* is the Freundlich parameter related to binding affinity and *m* is the sorbent’s heterogeneity index, different models were also fitted to the experimental results. The value of m ranges from 0 to 1. When *m* = 1, a system is considered to be entirely homogenous. The inverse dissociation constant *K_D_* is produced by the mean association constant *K*_0_, which functions as a function of *a* and *m* (Equations (2) and (3)). The model with the best fit is determined from the size of the correlation coefficients (R^2^) of the isotherm models. The kinetic data analysis results of both peptide and protein adsorption are presented in Table 1. When the data were examined, it was seen that peptide adsorption was compatible with the Langmuir model (R^2^ = 0.972), while the highest R^2^ value (0.984) for protein adsorption was reached in the Langmuir–Freundlich model. According to these results, it can be interpreted that the peptide adsorption to nanoMIPs was mostly one-to-one (homogeneous), but because the protein molecules were larger than the peptide, the adsorption shifted to a slightly more multilayered structure.

(2) $$K_{0}=a^{\frac{1}{m}}$$

(3) $$K_{D}=\frac{1}{K_{o}}$$

### 3.6. Selectivity and Cross-Reactivity Studies

**Figure 9:**
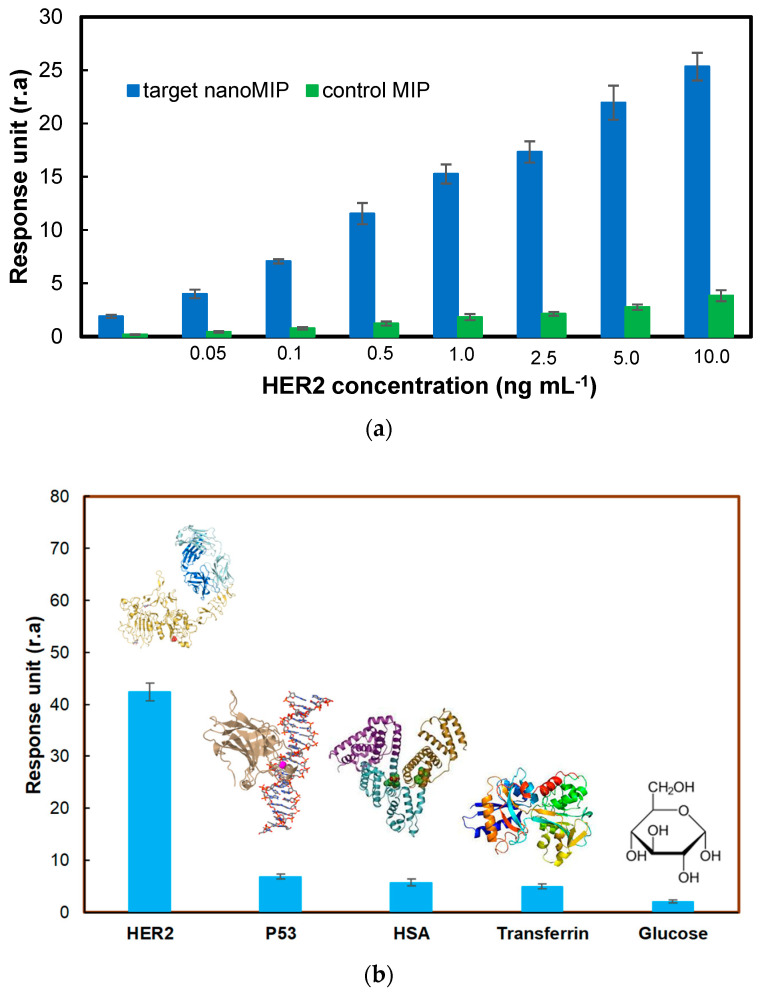
(**a**) Selectivity and (**b**) cross-reactivity studies using control nanoMIPs and reference molecules, respectively.

Control nanoMIPs were used to determine the selectivity of the target nanoMIPs. For this purpose, a completely different peptide sequence was chosen to be imprinted for obtaining the control polymer. Hence, the detection of HER2 protein in the concentration range of 0.01–10 ng mL^−1^ was examined on the sensor’s surface, which was prepared using the non-specific nanoMIPs. Figure 9a shows that the affinity of target nanoMIP against HER2 was much higher than that of control nanoMIP. The ability of control nanoMIPs to bind small amounts of HER2 was likely due to non-specific interactions. The selectivity coefficient (*k*) can be calculated as (Equation (4)):

(4) $$k=\frac{{RU}_{targetnanoMIP}}{{RU}_{controlMIP}}$$

In the selectivity studies performed at eight different HER2 concentrations, the average selectivity coefficient was found to be 8.56, indicating the high selectivity of the epitope-imprinted polymer toward HER2. 

A cross-reactivity study was also performed to determine the specificity of target nanoMIPs to HER2. To achieve this, the affinity of HER2-specific nanoMIPs was examined toward non-specific biomolecules, such as glucose, transferrin, HSA, and P53, at a fixed concentration of 100 ng mL^−1^. The non-specific binding of these biomolecules was quite low compared with the target molecule HER2, confirming the high specificity of the nanoMIPs against HER2 (Figure 9b). 

### 3.7. Serum Studies

**Figure 10:**
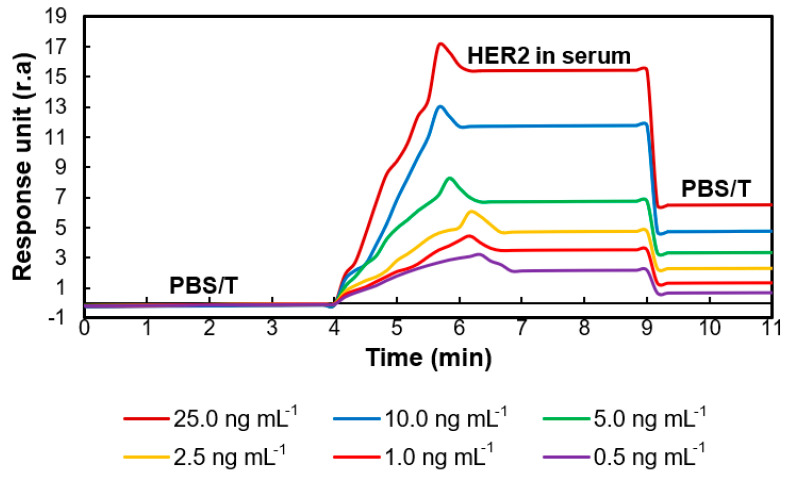
Sensograms obtained during the real-time detection of HER2 in human serum.

Assays were carried out using diluted human serum (1:10) with PBS buffer (pH: 7.4) to realize the potential application of the portable SPR in real sample analysis. The HER2 biomarker spiked in diluted human serum in the concentration range of 0.5–25.0 ng mL^−1^ was analyzed, and a gradual increase in SPR signals in a concentration-dependent manner confirmed the successful fabrication of the HER2-specific nanoMIP–SPR sensor (Figure 10). The LOD value was determined to be 11.6 pg mL^−1^. The amount of HER2 in the serum of healthy subjects varies between 2.0–15.0 ng mL^−1^. In breast cancer patients, this range is 15–75 ng mL^−1^ [15]; thus, the critical cut-off value to focus on is 15 ng mL^−1^, which is well above the LOD of our sensor. Of note, the higher LOD value in serum samples may be attributed to the interference of specific serum components, such as endogenous HER2, with the sensor’s surface [28]. 

Table 2 shows the LOD values of some biosensors reported in recent years for the detection of HER2 in serum samples. Accordingly, our nanoMIP–SPR provides comparable or better performance than those of the other studies [15,28,29,30,31]. Importantly, the newly developed sensor allows the real-time detection of HER2 without requiring any label, signal amplification agent, or redox marker. Along with these features, its rapid, cost-efficient, and portable nature may pave the way for the efficient and early diagnosis of HER2 in point-of-need.

## 4. Conclusions

In this study, a novel nanoMIP–SPR sensor was fabricated for the rapid and cost-effective detection of HER2 using the peptide-imprinted artificial receptors manufactured with a solid-phase synthesis technique. The nanoMIP receptors and the sensor fabrication steps were successfully characterized by employing various techniques, including DLS, TEM, EDX, FT-IR, fluorescence microscopy, CV, and SWV. The portable and label-free sensor allowed for the detection of the HER2 biomarker in a wide concentration range, with LODs of 3.8 pg mL^−1^ and 11.6 pg mL^−1^ in PBS and human serum samples, respectively. The synthetic receptors demonstrated a very high affinity (K_D_ = 215.08 pM), selectivity (8.56-fold higher sensing signals with HER2-specific nanoMIP in comparison with the control nanoMIP), and specificity toward HER2. The newly designed sensor provides great potential for further developments in the point-of-care diagnosis of breast cancer.

## Figures and Tables

**Table 1:** Calculated fitting parameters of the isotherm models.

Parameters for Peptide	Langmuir	Freundlich	Langmuir–Freundlich
N_t_	15.94	-	2103.36
a	0.56	4.62	0.0022
m	1	0.4646	0.4670
R^2^	0.972	0.960	0.955
K_D_	1.79 nM	0.037 nM	490.3 µM
**Parameters for Protein**	**Langmuir**	**Freundlich**	**Langmuir Freundlich**
N_t_	21.24	-	59.56
a	0.30	4.89	0.089
m	1.0	0.37	0.45
R^2^	0.938	0.979	0.984
K_D_	3.37 pM	0.0134 pM	215.08 pM

**Table 2:** A list of biosensors for HER2 detection in serum.

Sensor Type	Receptor Type	Serum Dilution Rate	Concentration Range (ng mL^−1^)	LOD (pg mL^−1^)	Ref
Optical-fiber	Ball resonator	1:10	0.001–128.0	3.7	[28]
SPR	Antibody	1:2	0.23–55.0	180.0	[15]
Electrochemical	Antibody	1:10	0.01–100.0	10.0	[29]
Electrochemical	Antibody	1:20	0.15–100.0	10.2	[30]
Electrochemical biosensor	Nanocomposite *	1:100	-	10.9	[31]
Portable SPR sensor	MIP	1:10	0.5–25.0	11.6	This work

* Nanocomposite of AMNFs@ZIF-67.

## Data Availability

The data presented in this study are available on request from the corresponding author.
